# Supplementary material for: LTA4H extensively associates with mRNAs and lncRNAs indicative of its novel regulatory targets
Source: PeerJ. 2023 Mar 10;11:e14875. doi: 10.7717/peerj.14875 (PMC10010175; doi:10.7717/peerj.14875)
Supplement: File S2 [file peerj-11-14875-s005.zip › Raw data files/Quality_control/FastqcView/LTA4H_input_1_fastqc/fastqc_report.html]

E743-input-1-mRNA\_all.fq.clean\_fq\_reverse FastQC Report


FastQC Report

Mon 16 Oct 2017  
E743-input-1-mRNA\_all.fq.clean\_fq\_reverse

## Summary

- Basic Statistics
- Per base sequence quality
- Per sequence quality scores
- Per base sequence content
- Per base GC content
- Per sequence GC content
- Per base N content
- Sequence Length Distribution
- Sequence Duplication Levels
- Overrepresented sequences
- Kmer Content

## Basic Statistics

| Measure | Value |
| --- | --- |
| Filename | E743-input-1-mRNA\_all.fq.clean\_fq\_reverse |
| File type | Conventional base calls |
| Encoding | Sanger / Illumina 1.9 |
| Total Sequences | 47442522 |
| Sequence length | 16-147 |
| %GC | 45 |

## Per base sequence quality

## Per sequence quality scores

## Per base sequence content

## Per base GC content

## Per sequence GC content

## Per base N content

## Sequence Length Distribution

## Sequence Duplication Levels

## Overrepresented sequences

| Sequence | Count | Percentage | Possible Source |
| --- | --- | --- | --- |
| TGTGTGCCTAATACATGCATGTTGAACGGGATGTAGCAATACATTCAGTA | 119723 | 0.25235378507070094 | No Hit |
| CTACGGGGTCTGACGCTCAGTGGAACGAAAACTCACGTTAAGGGATTTTG | 94782 | 0.19978280244039304 | No Hit |
| GTGGGCGTTGGAATATTGATGAGAGCTGCTCTTAGTACGAGAGGACCGGA | 71553 | 0.15082039694264146 | No Hit |
| GGCCGGTGTTCTTCAGGATCTGCTTGCTCACCATGGTTGTGGCCATTATC | 66403 | 0.13996515615253338 | No Hit |
| ACGGTGAAGAGACATGAGAGGTGTAGAATAAGTGGGAGGCCCCCGGCGCC | 52555 | 0.11077615140274372 | No Hit |
| GAGGTAGTGACGAAAAATAACAATACAGGACTCTTTCGAGGCCCTGTAAT | 50666 | 0.106794491237207 | No Hit |

## Kmer Content

| Sequence | Count | Obs/Exp Overall | Obs/Exp Max | Max Obs/Exp Position |
| --- | --- | --- | --- | --- |
| TGCCT | 8297095 | 1.8906972 | 5.187071 | 5 |

Produced by FastQC (version 0.9.5)
